# Believing in the Nobel Prize: Long-term Success After HLA-Identical Kidney Transplantation in 3 Monozygotic Twins Without any Induction or Maintenance Immunosuppression

**DOI:** 10.1097/TXD.0000000000001941

**Published:** 2026-04-08

**Authors:** Aylin Akifova, Ömer Ege Ömeroğlu, Fabian Halleck, Bilgin Osmanodja, Eva Vanessa Schrezenmeier, Friederike Bachmann, Diana Stauch, Nils Lachmann, Klemens Budde

**Affiliations:** 1 Department of Nephrology and Intensive Care, Charité-Universitätsmedizin Berlin, Berlin, Germany.; 2 Histocompatibility and Immunogenetics Laboratory, Centre for Tumor Medicine, Charité - Universitätsmedizin Berlin, Berlin, Germany.

## Abstract

**Background.:**

Kidney transplantation between HLA-identical monozygotic twins offers a unique chance for long-term graft survival without immunosuppression. Although favorable outcomes after withdrawal of maintenance immunosuppression have been reported, complete omission remains uncommon, and immunosuppressive strategies are not standardized across centers.

**Methods.:**

We report 3 HLA-identical monozygotic twin pairs who underwent kidney transplantation without induction or maintenance immunosuppression. Allograft function was monitored longitudinally, with follow-up durations of 22, 10, and 4 y, respectively. All patients had immediate and sustained excellent graft function with no rejection episodes or native disease recurrence during follow-up.

**Results.:**

Patient 1 was a 29-y-old man with chronic glomerulonephritis and had a largely uneventful course posttransplant. Patient 2 was a 61-y-old man who received his third graft from his monozygotic twin. Although highly sensitized because of previous transplants (calculated panel-reactive antibody = 62%), no desensitization or immunosuppressive therapy was administered, and graft function remained excellent without rejection. Patient 3 was a 43-y-old woman with IgA nephropathy, who was transplanted in high-risk cytomegalovirus constellation (donor [+]/recipient [–]). She developed a primary cytomegalovirus infection after cessation of 3-mo prophylaxis, which was successfully treated with valganciclovir.

**Conclusions.:**

This case series demonstrates that kidney transplantation between HLA-identical monozygotic twins can achieve excellent long-term graft survival without any induction or maintenance immunosuppression.

## INTRODUCTION

Kidney transplantation between HLA-identical twins represents a unique scenario in organ transplantation in which complete histocompatibility eliminates allorecognition and, accordingly, the need for long-term immunosuppression.^1^ Before the principles of histocompatibility were understood, early attempts to transplant xenografts between species or homografts from deceased donors into humans had failed, raising fundamental questions about why grafts were rejected. At that time, the immunological background for the failure of the experiments was still unknown, and allorecognition and rejection were understudied as concepts.^2^ This changed in 1954, when Murray et al performed the first successful human kidney transplantation between the identical twins, Ronald and Richard Herrick, in Boston.^3^ Before the operation, the team performed a full-thickness skin graft transplantation between the twins, a method then used to predict whether donor tissue would be accepted without an immune reaction (eg, rejection). The kidney transplant was surgically successful, renal function was immediate, and the graft survived 8 y, demonstrating that long-term graft survival could be achieved without any immunosuppression in cases of full immunological compatibility between donor and recipient, for example, in HLA-identical monozygotic twins.^3^

This landmark achievement prompted Murray et al^4^ to perform a series of additional kidney transplants among identical and nonidentical twins, siblings, and unrelated donors. Although all procedures were surgically successful, only grafts between monozygotic twins demonstrated consistent long-term survival, whereas most allografts between nonidentical individuals failed rapidly.^4^ These contrasting outcomes shifted attention away from surgical technique and toward the immunological basis of graft acceptance or rejection. During the following decade, the discovery of the HLA system and the recognition—by the mid-1960s—that donor-recipient HLA matching strongly influenced graft survival established the major histocompatibility complex compatibility between donor and recipient as the central determinant of transplant success.^5,6^ Together with the description of rejection and the underlying basic immunological principles, the first immunosuppressive drugs (eg, steroids and azathioprine) were developed, enabling the first successful transplants beyond monozygotic twins.^7,8^

Although early evidence from identical-twin transplants clearly demonstrated that long-term graft survival can be achieved without immunosuppression, the generalizability of this approach in modern clinical practice has remained surprisingly uncertain.^4^ The first systematic analysis of twin transplants performed between 1987 and 2006 suggested that immunosuppression may be unnecessary in HLA-identical-twin kidney transplantation. In the largest analysis of the Organ Procurement and Transplantation Network database, Krishnan et al^9^ identified 194 probable identical-twin transplants (no zygosity testing available) and found that recipients who were not maintained on immunosuppression had better renal function than those who continued immunosuppressive therapy. Those early observations implied that ongoing immunosuppression could be unnecessary—and potentially even detrimental—in this unique transplant population.^9^ This interpretation was reinforced by a joint UK-US registry analysis with up to 5-y follow-up, which similarly demonstrated that minimal immunosuppression or even complete avoidance of immunosuppression in identical-twin recipients is feasible and associated with excellent graft outcomes.^10^

Nevertheless, despite these observations, the consistent avoidance or cessation of immunosuppression remains uncommon in current clinical practice. Contemporary US registry data (Organ Procurement and Transplantation Network) confirm that identical-twin kidney transplantation remains an immunologically exceptional setting, with approximately 50% of recipients managed without maintenance immunosuppression.^1^ In this cohort of 143 kidney transplant recipients (2001–2017), Jorgensen et al^1^ report no differences in graft survival at 6 or 12 mo between patients with or without maintenance therapy, underscoring the feasibility of early immunosuppression withdrawal. However, these data are limited to short-term follow-up and do not address long-term immunologic quiescence.^1^ This gap is critical, as long-term outcome data under immunosuppression-free management remain scarce.

Recently, the Descartes Working Group in Europe analyzed 1351 publications and identified only 16 retrospective studies (a total of 5636 recipients) reporting outcomes in HLA-identical living-donor kidney transplantation under different immunosuppressive regimens. Six of these studies predate 1990, and only 3 included tacrolimus-based immunosuppression. Importantly, none of the studies used contemporary immunological risk assessment tools (eg, solid-phase HLA antibody assays), limiting the ability to stratify alloimmune risk or tailor immunosuppression to modern standards.^11^ Consequently, this systematic review concludes that there are no evidence-based recommendations or consensus on immunosuppression management in HLA-identical kidney transplant recipients.^9^ Notably, the evidence is even more limited regarding complete omission of immunosuppression: only a few reports describe very brief maintenance regimens or only induction/perioperative exposure to immunosuppression, and true immunosuppression-free protocols remain essentially scarce in this modern era of transplantation,^12,13^ despite the fact that Joseph Murray received the Nobel Prize for his landmark case of successful transplantation without any immunosuppression.^3^

## MATERIALS AND METHODS

In this case series, we report the long-term clinical outcomes of 3 pairs of HLA-identical-twin kidney transplants performed in the absence of induction or maintenance immunosuppression at our center. Clinical data were retrospectively collected from medical records, including recipient and donor characteristics, transplant course, laboratory parameters, and long-term graft outcomes. Donor and recipient kidney function was assessed by serial measurements of serum creatinine and estimated glomerular filtration rate, together with urine dipstick testing and extended urine analysis, including spot urine albumin-to-creatinine ratio as well as urinary erythrocyte and leukocyte counts. All patients were followed regularly according to institutional standards.

### Institutional Review Board Statement

Institutional review board approval was not required for this case series in accordance with local regulations. All patients provided written informed consent for the use and publication of pseudonymized clinical data.

## RESULTS

### Patient 1

A 29-y-old man with end-stage renal disease (ESRD) secondary to chronic glomerulonephritis had been on hemodialysis from 1997 until 2003. He had no prior history of kidney transplantation. In 2003, he received a kidney transplant from his HLA-identical monozygotic twin brother. The recipient’s medical history included renal hypertension, renal anemia, and a history of 10 pack-years of smoking (Table 1). The donor was in good health with no evidence of kidney disease or other comorbidities.

**TABLE 1.** Baseline and transplant characteristics of the patients

	Patient 1	Patient 2	Patient 3
Recipient age at transplant, sex	29-y-old, man	60-y-old, man	43-y-old, woman
Cause of ESRD (end-stage renal disease)	Chronic glomerulonephritis NOS	Glomerulonephritis NOS	Biopsy-proven IgA nephropathy (MEST-C score: M1, E0, S1, T2; 08/2012)
Recipient comorbidities	Arterial hypertension (renal) Nicotine abuse (10 pack-years) History of gastrointestinal bleeding Renal anemia	Cardiovascular Arterial hypertension Left ventricular hypertrophy Recurrent atrial fibrillation Nicotine abuse (20 pack-years) Hepatology/ Gastroenterology Chronic hepatitis C infection • Antiviral treatment: sofosbuvir + daclatasvir + ribavirin for 3 mo (December 2024) • No detectable HCV-RNA (May 2015) Corpus gastritis with *Helicobacter pylori* Serology: hepatitis A, C, E Mallory–Weiss lesion Cholecystolithiasis Endocrinology/metabolic disorders Type IIa non–insulin-dependent diabetes mellitus with polyneuropathy since 2008 Thyroidectomy and parathyroidectomy in 2001 Urology Benign prostatic hyperplasia Right orchiectomy (2001) Neurology Restless legs syndrome Oncology Differentiated adenocarcinoma of the sigmoid colon February 2024 • Pathology: pT2 pN0 (0/11) L1 V0 Pn0, M0, R0 • Local disease, complete resection • Surgical history (2024): ○ Midline laparotomy ○ Jejunal segment resection ○ Left colon resection, including left flexure and sigmoid colon Reconstruction: transverse colon to rectum anastomosis (end-to-end)	Renal anemia, renal hypertension, and metabolic acidosis Secondary hyperparathyroidism (renal osteopathy); Nicotine abuse (5 pack-years) Hyperlipoproteinemia Hyperuricemia Thrombophilia with lupus anticoagulant, elevated factor VIII levels, and heterozygous APC resistance (Factor V Leiden), as well as Prothrombin G20210A mutation, MTHFR variant, PAI-1, tPA, and FSAP abnormalities
Dialysis modality and duration	Hemodialysis October 1997–February 2003 (5 y and 4 mo)	Hemodialysis prior the first kidney transplantation: December 1985–December 1988 (3 y) Between the first and second kidney transplantation: April 1996–July 2000 (4.25 y) Between the second graft loss and third, most recent transplantation: December 2005–June 2015 (9.5 y)	CAPD: February 2016–November 2016 HD: November 2016–January 2017 CCPD: January 2017–November 2021 Total duration of dialysis (5 y and 8 mo)
Previous transplants and causes of graft loss	None	1st kidney transplant (November 1988, right iliac fossa) Graft failure and transplant nephrectomy: April 1996 2nd kidney transplant (July 2000, left iliac fossa) Acute rejection: Banff IIa in October 2000 Biopsy in October 2005: membranoproliferative glomerulopathy Graft failure: December 2005 Transplant nephrectomy: October 2008	None
cPRA% at most recent transplantation	0	62%	0
Transplant year	2003	2015	2021
CMV^–^/ EBV high-risk constellation	No	No	Yes, for CMV
Best creatinine after transplant	1.04 mg/dL	0.97 mg/dL	0.89 mg/dL
Infectious complications	None, COVID infection in 2022		Nitrite positive UTI in the second postoperative week, treated with ampicillin/sulbactam Primary CMV infection in high-risk constellation after stop of regular prophylaxis
Renal complications	Transient moderate proteinuria early posttransplant, spontaneously resolved	Prerenal AKI stage II in the context of an anastomotic insufficiency April 2024 Maximum creatinine rise: 2.4 mg/dL	Transient mild proteinuria: resolved
Kidney allograft biopsy	Not performed	Not performed	mild acute tubular epithelial injury and mild global glomerulosclerosis (4/17 glomeruli). No signs of rejection, no recurrence of IgA nephropathy
Pregnancy history	Not applicable (male)	Not applicable (male)	no pregnancies posttransplant, 1 pregnancy before transplantation
Donor status at transplantation	29-y-old, man Serum creatinine: 1.28 mg/dL No proteinuria Normal blood pressure	60-y-old, man Serum creatinine: 0.63 mg/dL No proteinuria	43-y-old, woman Serum creatinine: 0.90 mg/dL No proteinuria
Donor comorbidities	none	Arterial hypertension Nicotine abuse History of thyroid gland surgery NOS Subcutaneous benign lipoma No malignancies	Arterial hypertension Nicotine abuse (15 pack-years) Thrombophilia with lupus anticoagulant, elevated factor VIII levels, and heterozygous APC resistance (Factor V Leiden), as well as Prothrombin G20210A mutation, MTHFR variant, PAI-1, tPA, and FSAP abnormalities
Donor kidney function at last follow-up	Serum creatinine: 1.14 mg/dL No proteinuria Normal blood pressure	Serum creatinine: 0.75 mg/dL No proteinuria Well-controlled hypertension (present pretransplant)	Serum creatinine: 0.79 mg/dL No proteinuria Well-controlled hypertension (present pretransplant)

AKI, acute kidney injury; APC, activated protein C; CAPD, continuous ambulatory peritoneal dialysis; CCPD, continuous cycling peritoneal dialysis; CMV, cytomegalovirus; cPRA, calculated panel-reactive antibody; EBV, Epstein–Barr virus; ESRD, end-stage renal disease; FSAP, factor VII activating protease; HCV, hepatitis C virus; HD, Hemodialysis; MEST-C, histopathological scoring system: M, mesangial hypercellularity, E, endocapillary hypercellularity, S, segmental glomerulosclerosis, T, tubular atrophy/interstitial fibrosis, C, crescents; MTHFR; methylenetetrahydrofolate reductase; NOS, not otherwise specified; PAI-1, plasminogen activator inhibitor-1; tPA, tissue plasminogen activator; UTI, urinary tract infection.

**FIGURE 1.**
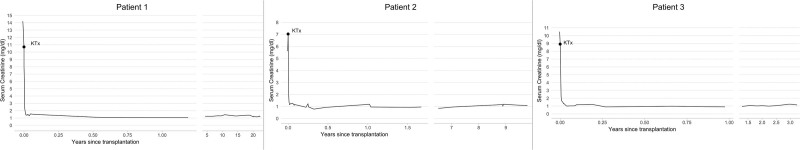
Serum creatinine curves of patients 1, 2, and 3 throughout the follow-up period.

Given the HLA-identical-twin donation, no immunosuppressive therapy—neither for induction nor for maintenance—was administered. The transplantation was technically uneventful, and the graft function was immediate, with creatinine improving to 1.04 mg/dL in early 2004 and remaining stable during the subsequent 22.5 y (Figure 1).

Through follow-up, the patient experienced no infectious or immunological complications, except for a COVID-19 infection in 2022, which was resolved completely without sequelae. Screening for HLA- and non-HLA-antibodies, such as angiotensin II type 1 receptor antibodies and endothelin type A receptor antibodies, remained negative throughout the follow-up period. Both donor and recipient preserved an excellent kidney function, and laboratory values and blood pressure were within normal ranges at all routine outpatient visits, and both reported overall well-being.

### Patient 2

A 61-y-old man received his third renal transplant from his HLA-identical twin in 2015. Historically, he had developed ESRD due to glomerulonephritis requiring renal replacement therapy (RRT) with hemodialysis in 1985. He received his first kidney transplant from a deceased donor in 1988 and a second deceased donor kidney in 2000; however, both previous allografts were lost after a higher grade acute cellular rejection and advanced fibrosis after 5 and 7 y, respectively. Transplant nephrectomy of both allografts was indicated after intragraft thrombotic complications along with resistant hypertension and the patient was maintained on hemodialysis for another 10 y before his most recent transplantation. Further medical history revealed immunosuppression-related complications such as renal cell adenocarcinoma of advanced stage, which indicated a nephrectomy of his native kidneys, a chronic hepatitis C infection, successfully treated with direct-acting antiviral therapy, a helicobacter pylori-associated gastritis, and a high cardiovascular risk profile, including 20 pack-years of smoking, atrial fibrillation, and arterial hypertension. Additionally, the mandatory immunological crossmatch test before transplantation revealed a calculated panel-reactive antibody of 62%, indicating alloimmunization from the previous transplants and resulting in a much more limited donor pool.

However, neither induction nor maintenance immunosuppression was initiated regarding the full immunological compatibility with his HLA-identical-twin donor. The transplant of his twin-donor kidney was successfully performed in 2015, followed by an immediate graft function, and a nadir serum creatinine of 1.16 mg/dL was reached 5 d posttransplant. After an uneventful surgery, no relevant complications occurred. The donor had a history of well-controlled hypertension and thyroid gland surgery and recovered well after surgery and maintained good kidney function after donation (Table 1).

In the later posttransplant period, the patient developed a differentiated adenocarcinoma of the sigmoid colon. Pathology revealed a localized disease, and complete surgical resection was performed successfully. The postoperative course was complicated by prerenal acute kidney injury stage II in the context of an anastomotic insufficiency with a maximum serum creatinine rise to 2.4 mg/dL, which gradually improved with supportive management, and kidney function quickly returned to baseline. Oncological follow-up demonstrated no signs of recurrence, and the patient has experienced no additional complications thus far. Additionally, there was no evidence of HLA or non-HLA antibodies directed against the most recent graft during the transplant period.

Overall, kidney graft function remained stable during 10 y after transplant, and both donor and recipient are in good health according to the last follow-up in 2025, with well-controlled blood pressure and fully preserved kidney function (Figure 1).

### Patient 3

A 43-y-old woman was hospitalized because of acute kidney dysfunction of unknown origin. Further evaluation and laboratory work-up suggested an underlying immunological kidney disease, and a native kidney biopsy finally confirmed an IgA nephropathy (IgAN).

The histological examination revealed a highly active mesangioproliferative glomerulonephritis, which was scored as M1, E0, S1, and T2 according to the Oxford classification for IgAN.^14^ In <4 y, the patient progressed into ESRD and RRT was started with a peritoneal dialysis system initially. However, multiple complications occurred within 9 mo, and at last, after catheter-related peritonitis, the patient was switched to hemodialysis via a tunneled dialysis catheter for more effective RRT during the recovery period but was then returned to continuous cycling peritoneal dialysis.

After 5 y of dialysis, the patient received a kidney transplant from her monozygotic twin sister in 2021 without any induction or maintenance immunosuppression. The recipient had immediate graft function without renal complications, with a nadir creatinine of 0.89 mg/dL. Due to a high-risk constellation regarding cytomegalovirus (CMV)-status (donor [+]/ recipient [–]), the patient received a 3-mo prophylaxis therapy with valganciclovir. However, a few weeks after the end of prophylaxis, the patient presented with night sweats, and a positive blood polymerase chain reaction test confirmed a primary CMV infection. Immediate treatment with valganciclovir was started, and the infection resolved quickly without further symptoms or complications. The patient had no further reactivation episodes during the follow-up period. After 3 y of a stable clinical course, a routine evaluation during an outpatient visit revealed transient moderate proteinuria, which triggered a clinical biopsy to rule out possible IgAN recurrence; however, there were neither signs of IgA deposition nor other abnormal histological findings. Furthermore, the proteinuria resolved rapidly without impacting the renal function. There was no evidence of HLA- or non-HLA antibodies during the transplant period (Table 1). The patient experienced no pregnancies and miscarriages after transplantation and had 1 daughter from an uneventful pregnancy at the age of 28 y before transplantation. Both donor and recipient have excellent kidney function and overall health status 4 y after transplantation (Figure 1).

## DISCUSSION

Kidney transplantation between HLA-identical twins remains the most immunologically favorable context in transplantation, offering a unique setting for excellent long-term outcomes without the need for immunosuppressive therapy. In our report, we present 3 pairs of monozygotic twin transplants performed without any induction and maintenance immunosuppression. All recipients demonstrated immediate and perfect long-term graft function without evidence of rejection. Our findings contribute to the limited literature showing that durable immunosuppression-free graft survival is achievable in carefully selected identical-twin pairs.

Despite this immunologic “best-case scenario” in monozygotic twin kidney transplantation, which was awarded the Nobel Prize in 1990, many centers continue to use some immunosuppressive therapy because of potential theoretical concerns, such as perioperative immune activation, recurrence of native kidney disease, and rare cases of rejection due to non-HLA mechanisms.^11^ Some clinicians administer perioperative steroids or other induction immunosuppressants to prevent theoretical nonalloimmune immune activation from ischemia/reperfusion injury.^15,16^ However, there is no evidence that these transient responses cause rejection or long-term graft damage, making routine perioperative immunosuppression unproven in this setting.

Beyond these early transplantation-related concerns, the primary long-term risk in this population is recurrence of the patient’s original kidney disease, together with the potential long-term risks of the donor. Conditions such as focal-segmental glomerulosclerosis, IgAN, membranous nephropathy, and others can recur independently of alloimmune mechanisms. Importantly, zero-HLA-mismatch is a recognized risk factor for IgAN recurrence.^17^ Although studies show that recurrence is possible, maintenance immunosuppression has not been proven to prevent recurrence in HLA-identical-twin transplants and carries additional risks and side effects.^18^ Therefore, the risk of disease recurrence alone does not justify maintenance therapy, although immunosuppression and/or other treatments can be initiated if recurrence appears.

Concerns also exist about specific immune injury that could result from non-HLA disparities, epigenetic drift, or innate immunity activation, which can harm the allograft. However, these events are very uncommon, and no evidence supports routine immunosuppression to prevent them in monozygotic twin transplants.^19^ Furthermore, monozygotic twins usually share the same placenta and frequently exchange blood during pregnancy, supporting the development of true tolerance to even minor non-HLA disparities.^20-22^

Consequently, the rationale for avoiding complete immunosuppression in HLA-identical-twin kidney transplantation is strong, and immunosuppressive therapy carries well-documented risks—including infection, malignancy, metabolic complications, and drug-related nephrotoxicity, for example, with the predominantly used calcineurin inhibitors.^23-26^ In the setting of monozygotic twin transplantation, the primary target of these drugs—alloimmune rejection—is nearly absent, making the risk–benefit balance strongly favorable for complete omission. Additionally, successful pregnancies after HLA-identical-twin kidney transplantation have been reported. Murray et al described that Edith Helm—who received a kidney from her monozygotic twin sister Wanda and remains the longest-surviving kidney transplant recipient with a functioning graft for approximately 55 y—completed 2 pregnancies posttransplant without loss of graft function and without immunosuppressive therapy. Given that pregnancy itself represents a significant immunological challenge and is typically associated with additional risks in immunosuppressed transplant recipients, this observation further supports the feasibility of durable, long-term immunosuppression-free outcomes in this unique setting.^27^ Thus, it is somewhat surprising that most identical-twin transplant recipients receive immunosuppression, suggesting a degree of caution despite well-established immunological principles described >70 y ago.

This practice may be explained by the desire to maximize the success of a precious graft, the assumption that short-term immunosuppression is associated with only minor adverse effects, and the concern of omitting a potentially relevant therapeutic component. Indeed, early transplant experiences highlighted how unusually limited immunosuppressive regimens appeared when compared with current standard protocols, reinforcing a sense of caution among transplant teams.

Our case series demonstrates that long-term graft survival without induction or maintenance immunosuppression is feasible in HLA-identical twins, even in recipients who are highly sensitized patients due to previous transplants, such as patient 2 in our report. Even in the presence of 62% calculated panel-reactive antibodies, it is important to note that none of these HLA antibodies is directed against self-antigens identical to the HLA antigens of his identical-twin brother. These findings fully support Murray´s discovery^3^ and reinforce that complete omission is not only safe but may also provide superior long-term outcomes by eliminating the cumulative toxicity and complications associated with lifelong immunosuppressive therapy.

Despite the absence of alloimmune risk, identical-twin transplantation does not eliminate all posttransplant complications. In our cohort, 1 patient with a high-risk CMV constellation (D^+^/R^–^) developed primary CMV infection shortly after discontinuation of prophylaxis. Although standard-of-care prophylaxis for CMV high-risk recipients is 6 mo at our center and in accordance with current guidelines, prophylaxis was limited to 3 mo in this case, based on individualized clinical decision-making to avoid complete immunosuppression. The infection resolved fully with treatment, but this case highlights that donor-derived infections remain relevant even in the complete absence of immunosuppression. The second patient developed renal cell carcinoma 8 y after the first transplant and colon cancer 9 y after his third transplant at the age of 70. It is tempting to speculate that cumulative immunosuppression from the previous 2 grafts and uremia might have contributed to the development of kidney and colon cancer in this patient. These complications may arise independently of HLA compatibility, and standard surveillance, prophylaxis, and clinician awareness remain essential for posttransplant care.

Potential genetic risk for donors should also be considered in monozygotic twin transplantation, particularly when ESRD is caused by glomerulonephritis. Related living kidney donors have been shown to carry an increased long-term risk of kidney disease compared with unrelated donors, particularly in the context of familial or genetic renal disorders.^28^ In monozygotic twins, this risk may be even higher given the shared genetic background. Therefore, careful donor evaluation, including extended assessment of renal function, is essential before donation. In our cohort, all donors demonstrated normal renal function and no evidence of proteinuria or persistent hematuria at the time of donation but also during follow-up.

A key limitation of our series is that zygosity testing was not performed; however, we are confident that the donors and recipients are monozygotic based on fully HLA-identical profiles and a well-documented similarity in photographs since early childhood. Registry analyses and recent expert consensus emphasize that although same-sex, same-blood-type, and similar physical characteristics suggest monozygosity, clinical assumptions alone cannot always replace genetic confirmation. Formal testing using short tandem repeat profiling or single nucleotide polymorphism–based methods could therefore provide even better reassurance that donors and recipients are truly genetically identical today.^1^

We conclude that eligible HLA-identical siblings, if in good health and without medical or psychological barriers to donation, should be favorably evaluated as potential donors, given the high likelihood of long-term, complication-free graft survival without immunosuppression.

## References

[1] Jorgensen DR Wu CM Hariharan S. Epidemiology of end-stage renal failure among twins and diagnosis, management, and current outcomes of kidney transplantation between identical twins. Am J Transplant. 2020;20:761–768. 31595679 10.1111/ajt.15638

[2] Morris PJ. Transplantation—a medical miracle of the 20th century. N Engl J Med. 2004;351:2678–2680. 15616201 10.1056/NEJMp048256

[3] Merrill JP Murray JE Harrison JH. Successful homotransplantation of the human kidney between identical twins. J Am Med Assoc. 1956;160:277–282. 13278189 10.1001/jama.1956.02960390027008

[4] Murray JE Merrill JP Harrison JH. Kidney transplantation between seven pairs of identical twins. Ann Surg. 1958;148:343–359. 13571912 10.1097/00000658-195809000-00004 PMC1450810

[5] Bodmer W. A historical perspective on HLA. Immunother Adv. 2023;3:ltad014. 37636243 10.1093/immadv/ltad014 PMC10449411

[6] Opelz G Döhler B. Effect of human leukocyte antigen compatibility on kidney graft survival: comparative analysis of two decades. Transplantation. 2007;84:137–143. 17667803 10.1097/01.tp.0000269725.74189.b9

[7] Starzl TE. History of clinical transplantation. World J Surg. 2000;24:759–782. 10833242 10.1007/s002680010124 PMC3091383

[8] Halloran PF. Immunosuppressive drugs for kidney transplantation. N Engl J Med. 2004;351:2715–2729. 15616206 10.1056/NEJMra033540

[9] Krishnan N Buchanan PM Dzebisashvili N. Monozygotic transplantation: concerns and opportunities. Am J Transplant. 2008;8:2343–2351. 18808409 10.1111/j.1600-6143.2008.02378.x PMC2678894

[10] Kessaris N Mukherjee D Chandak P. Renal transplantation in identical twins in United States and United Kingdom. Transplantation. 2008;86:1572–1577. 19077892 10.1097/TP.0b013e31818bd83d

[11] Pérez-Sáez MJ Montero N Oliveras L; ERA-EDTA-Descartes working group. Immunosuppression of HLA identical living-donor kidney transplant recipients: a systematic review. Transplant Rev. 2023;37:100787. 10.1016/j.trre.2023.100787 37657355

[12] Yakubu I Haririan A Bartlett S. Successful renal transplantation between identical twins with very brief immunosuppression. Case Rep Transplant. 2018;2018:9842893. 30079258 10.1155/2018/9842893 PMC6040249

[13] Sánchez-Escuredo A Barajas A Revuelta I. Kidney transplant from a living monozygotic twin donor with no maintenance immunosuppression. Nefrologia. 2015;35:358–362. 26306949 10.1016/j.nefro.2015.06.006

[14] Cattran DC Coppo R Cook HT; Working Group of the International IgA Nephropathy Network and the Renal Pathology Society. The Oxford classification of IgA nephropathy: rationale, clinicopathological correlations, and classification. Kidney Int. 2009;76:534–545. 19571791 10.1038/ki.2009.243

[15] Salvadori M Rosso G Bertoni E. Update on ischemia-reperfusion injury in kidney transplantation: pathogenesis and treatment. World J Transplant. 2015;5:52–67. 26131407 10.5500/wjt.v5.i2.52 PMC4478600

[16] Tang Q Dong C Sun Q. Immune response associated with ischemia and reperfusion injury during organ transplantation. Inflamm Res. 2022;71:1463–1476. 36282292 10.1007/s00011-022-01651-6 PMC9653341

[17] McDonald SP Russ GR. Recurrence of IgA nephropathy among renal allograft recipients from living donors is greater among those with zero HLA mismatches. Transplantation. 2006;82:759–762. 17006322 10.1097/01.tp.0000230131.66971.45

[18] Rao Z Huang Z Song T. A lesson from kidney transplantation among identical twins: case report and literature review. Transpl Immunol. 2015;33:27–29. 26189977 10.1016/j.trim.2015.07.004

[19] Jethwani P Rao A Bow L. Donor-recipient non-HLA variants, mismatches and renal allograft outcomes: evolving paradigms. Front Immunol. 2022;13:822353. 35432337 10.3389/fimmu.2022.822353 PMC9012490

[20] Brent L. The discovery of immunologic tolerance. Hum Immunol. 1997;52:75–81. 9077556 10.1016/S0198-8859(96)00289-3

[21] Brent L. Transplantation tolerance—a historical introduction. Immunology. 2016;147:267–268. 26694700 10.1111/imm.12567 PMC4754606

[22] Owen RD. Immunogenetic consequences of vascular anastomoses between bovine twins. Science. 1945;102:400–401. 17755278 10.1126/science.102.2651.400

[23] Agrawal A Ison MG Danziger-Isakov L. Long-term infectious complications of kidney transplantation. Clin J Am Soc Nephrol. 2022;17:286–295. 33879502 10.2215/CJN.15971020 PMC8823942

[24] Paccagnella C Andreola S Gambaro A. Immunosuppressive therapy-related cardiovascular risk factors in renal transplantation: a narrative review. Cardiorenal Med. 2025;15:209–228. 39956105 10.1159/000542378

[25] Al-Adra D Al-Qaoud T Fowler K. *De novo* malignancies after kidney transplantation. Clin J Am Soc Nephrol. 2022;17:434–443. 33782034 10.2215/CJN.14570920 PMC8975024

[26] Rostaing L Jouve T Terrec F. Adverse drug events after kidney transplantation. J Pers Med. 2023;13:1706. 38138933 10.3390/jpm13121706 PMC10744736

[27] Murray JE. Edith Helm (April 29, 1935-April 4, 2011): the world’s longest surviving transplant recipient. Am J Transplant. 2011;11:1545–1546. 21718444 10.1111/j.1600-6143.2011.03607.x

[28] Mjøen G Hallan S Hartmann A. Long-term risks for kidney donors. Kidney Int. 2014;86:162–167. 24284516 10.1038/ki.2013.460

